# Comparative analysis morphology, anatomical structure and transcriptional regulatory network of chlorophyll biosynthesis in *Oryza longistaminata*, *O. sativa* and their F_1_ generation

**DOI:** 10.7717/peerj.12099

**Published:** 2021-09-06

**Authors:** Zhihang Hu, Xinyu Chen, Liexiang Huangfu, Shaobo Shao, Xiang Tao, Lishuang Song, Wenzhi Tong, Chuan-Deng Yi

**Affiliations:** 1 Jiangsu Key Laboratory of Crop Genetics and Physiology/Key Laboratory of Plant Functional Genomics of the Ministry of Education/Jiangsu Key Laboratory of Crop Genomics and Molecular Breeding, Agricultural College of Yangzhou University, Yangzhou, China; 2 College of Bioscience and Biotechnology, Yangzhou University, Yangzhou, China; 3 Jiangsu Co-Innovation Center for Modern Production Technology of Grain Crops, Yangzhou University, Yangzhou, China

**Keywords:** Morphology, Anatomical structure, Chlorophyll, Transcriptional regulatory, *Oryza sativa*, *Oryza longistaminata*

## Abstract

*Oryza longistaminata*, a perennial wild species, is widely distributed in the African continent. It has strong tolerance to biotic and abiotic stresses, and high biomass production on poor soils. Chlorophyll biosynthesis is important for photosynthesis in rice. However, the chlorophyll biosynthesis and related gene profiles of *O. longistaminata* and its descendants remained unclear. Here, the F_1_ generation of *O. sativa* and *O. longistaminata* were obtained. Then, the comparative analysis morphology, anatomical structure, and transcriptional regulatory networks of chlorophyll biosynthesis were detected and analyzed. Results showed that the F_1_ generation has obvious long awn, similar with that of the male parent. The purple color of the long awn is different from that of the male parent. Microstructural results showed that the flag leaves of F_1_ have large mesophyll cell gaps in the upper- and lower-positions, small mesophyll cell gaps in the middle position, and more chloroplasts. Increased chlorophyll content was also observed in the F_1_ generation. In the lower-position flag leaves, the total chlorophyll contents of F_1_ were 1.55 and 1.5 times those of *O. sativa* and *O. longistaminata*, respectively. *POR*, *MgCH* and *HEMA1* showed higher expression levels than the other related genes selected in the chlorophyll biosynthesis pathway. The *HEMA1* expression level in the middle-position flag leaves of *O. longistaminata* was the highest, and it was 2.83 and 2.51 times that of *O. sativa* and F_1_, respectively. The expression level of *DVR* gene in lower-position flag leaves of F_1_ were 93.16% and 95.06% lower than those of *O. sativa* and *O. longistaminata*, respectively. This study provided a potential reference for studying the photosynthesis and heterosis utilization of *O. longistaminata*.

## Introduction

Rice (*Oryza sativa* L.) is the one of most important crops and essential daily foods for more than half of the worldwide population. It is a model plant species for research on physiology, genetics, evolution and domestication of higher plants (Goff et al., 2002; Yu et al., 2002). Rice is also a model plant for studying photosynthesis and chloroplasts (Sage & Sage, 2009; Ermakova et al., 2020). *O. longistaminata* is a perennial cross-pollinated plant that originated in tropical Africa. As the most primitive ancestor of cultivated rice, *O. longistaminata* accumulated abundant beneficial genes, such as resistance to disease and insect, resistance to adversity, male sterility and strong regenerative ability (Park et al., 2010).

In higher plants, chlorophylls are important for photosynthesis in terms of harvesting light energy and as a transfer center. Chlorophylls are composed of chlorophyll *a*, *b*, *c* and *d* (Beale, 2005). Numerous reports could be found on the factors affecting plant photosynthesis in rice (Stenbaek & Jensen, 2010). Sasaki and Ishii compared photosynthetic rates in individual leaves among *Japonica* varieties (Sasaki & Ishii, 1992). Cook and Evans detected and analyzed the physiological aspects about the domestication and improvement of *Indica* and *Japonica* varieties with other species of *Oryza* (Cook & Evans, 1983). Crossing between cultivated rice (*Oryza sativa*) and wild rice species (*Oryza rufipogon*) could increase leaf photosynthesis (Masumoto et al., 2004). Wang and his colleagues reported that the contents of chlorophyll positively affected the biomass, photosynthetic rate, and grain yield in rice (Wang et al., 2008). Understanding the chlorophyll biosynthesis and its influential factors could help increase the accumulation of light assimilates and ultimately increase rice yield.

In this study, *O. longistaminata*, *O. sativa* and their F_1_ generation were selected as experimental materials. The morphology and anatomical structure of the plant and the flag leaf were observed. The chlorophyll contents and photosynthetic parameter, net photosynthetic rate (*Pn*), stomatal conductance (*Gs*), intercellular CO_2_ concentration (*Ci*) and transpiration rate (*Tr*), in the different of positions of the flag leaf were detected and analyzed. Real-time fluorescence quantitative polymerase chain reaction (RT-qPCR) was used to determine the expression profiles of chlorophyll biosynthesis-related genes. The results of this work provided a potential theoretical basis for research on photosynthetic characteristics and molecular mechanism among African wild rice, cultivated rice, and their F_1_ generation.

## Materials and Methods

### Plant materials

In June 2020, the plants of *O. longistaminata* (IRGC 104977), originated from Kenya, the elite indica variety of *O. sativa* ‘Huanghuazhan’ and their F_1_ generation were planted in Key Laboratory of Plant Functional Genomics of the Ministry of Education, College of Agriculture, Yangzhou University. The rice plants were routine water and fertilizer management. F_1_ plants were derived from a cross between Asian cultivated rice ‘Huanghuazhan’ as the female parent and African wild rice *O. longistaminata* as the male parent.

The F_1_ generation was obtained by distant hybridization. The flowering time of *O. longistaminata* was advanced by shading treatment. As *O. longistaminata* is a short-day crop, the flowering period is later than that of ‘Huanghuazhan’ under normal light conditions. Shading treatment was carried out on *O. longistaminata* from tillering stage from 5:00 P.M. to 9:00 A.M. of the next day to synchronize the flowering period of both parents. When the parents and F_1_ generation were in the same heading stage, the normal light condition was restored. At the heading stage, the flag leaves were collected, immediately frozen in liquid nitrogen, and stored at −80 °C for further experiments. Three biological replicates were performed for each sample.

### Photosynthetic parameters detection

At 9:00 A.M., the photosynthetic parameters of the rice flag leaves were measured using an LI-6400XT portable photosynthetic apparatus (LI-COR, Lincoln, NE, USA). Data of net photosynthetic rate (Pn), stomatal conductance (Gs), intercellular CO_2_ concentration (Ci) and transpiration rate (Tr) were collected as previously described in Jiang et al. (2014). Specifically, the air flow rate was 500 μmol·s^−1^, the irradiance was 700 μmol·m^−2^·s^−1^, the temperature was 20 °C, the relative humidity was 70%, the CO_2_ concentration was 400 µmol·mol^−1^, and the leaf chamber area was 11 cm^2^ (Jiang et al., 2014). Three different positions, namely, upper, middle and lower, of the flag leaves, were selected. Three biological replicates were performed for each sample.

### Determination of chlorophyll content

The chlorophyll content was determined *via* extraction of acetone and anhydrous ethanol. Rice flag leaf samples (0.1 g) were macerated and ground in 10 mL test tube of 95% ethyl alcohol in a mortar and the chlorophyll was extracted in the liquid after filtration. Then add acetone: ethanol 1:1 mixture, seal and extract in the dark until the leaves turn white completely. The absorbance was measured at the wavelengths of 649 nm and 665 nm by PE-2100 atomic absorption spectrophotometer (PerkinElmer, Waltham, MA, USA). The contents of Chl *a* and Chl *b* were calculated using the equations of (Lichtenthaler, 1987). Three biological replicates were performed for each sample.

### Microstructural observation

The upper-, middle- and lower-positions of the flag leaves were collected from the *O. sativa*, *O. longistaminata* and their F_1_ generation. These samples were immediately soaked in a fixative containing 2.5% glutaraldehyde in phosphate buffer (pH 7.2) at 4 °C for 24 h. Microstructural observation was performed following Chen’s method with some modifications (Chen et al., 2019). Briefly, afterwards, the rice flag leaf samples were rinsed, dehydrated, substituted, embed, and polymerized to generate the resin-embed sample, which was then cut longitudinally or transversely into 1 mm slices using an ultramicrotome (Ultracut R, Leica, Germany). Data were collected as previously described in Chen et al. (2019).

### Extraction of RNA and synthesis of cDNA

Total RNA samples of ‘Huanghuazhan’, *O. longistaminata* and their F_1_ were extracted from rice flag leaves by RNA Simple Total RNA Kit (Tiangen Company, Beijing). The RNA concentrations were determined by micro-ultraviolet detector Nano-Drop (Thermo Scientific, Wilmington, DE, USA). The total RNA of rice flag leaves with different materials was reverse-transcribed into cDNA by the reverse transcription Kit (TaKaRa Company, Dalian, China). Three biological replicates were performed for each sample.

### RT-qPCR analysis

RT-qPCR reaction was performed in according with the instructions in the ACEQ qPCR SYBR Green Master Mix kit (Nanjing Vazyme Biotechnology Co., Ltd., Nanjing, China). The rice *Actin* gene was used as the reference gene, and the amplification primers were Actin-F and Actin-R (Table 1). The reaction system was 20 μL, including 10 μL ACEQ qPCR SYBR Green Master Mix, 0.4 μL 50 × Rox Reference Dye 1B, 2 μL diluted cDNA, 0.4 μL positive and reverse fluorescence quantification primers and 6.80 μL ddH_2_O. The reaction conditions were as follows: the first stage was pre-denaturation at 95 °C for 5 min, the second stage includes 40 cycles, denaturation at 95 °C for 10 s, annealing at 60 °C for 30 s; Finally, it was extended at 65 °C for 15 s. The fluorescent quantitative detection primers were designed by Primer Premier 6.0 software as shown in Table 1. The relative expression ratio was calculated by the ΔΔ^Ct^ method (Pfaffl, 2001). Three biological replicates were performed for each sample.

**Table 1:** RT-qPCR primer sequence.

Gene name	Forward primer sequence (5′–3′)	Reverse primer sequence (5′–3′)
*GLURS*	GGTGAACTTTGTCAAGCACTAG	CTTTGTGTATGAGGACTACGGT
*ALAD*	GGCATGGGCTTCTTGATGAGG	CCAGAGCAACATCCGTGTAGAC
*PBGD*	ACATATTTACCAGAAGGCACGA	TGAGATTGTCTACGCAGAGAAG
*UROD*	TGCGAGCGACATCCTTCATT	ATGTTCATCCCAGGAAGCGG
*CPOX*	AAGCACCGTAATGAGCGTCG	TCAGTTGTTGCCATGCCTTGT
*POR*	TTGAGACCAACCGCACAACA	GACTCGATTGGGGGCATGAG
*DVR*	CAGGTTCATCAAGGTGCCGAT	CGTCATCTCGTCGCTGTACTC
*HCAR*	GCAGGAAGCAAGACATGGATGA	GCATTGGAGCAAGCCTGTCA
*CHLG*	GGGCACTGTTGTTAGCAGGG	GCCAATGTAGCTCGCACCAA
*MgCH*	AAGATGGTTGCCGAACTGGATG	ATGTCCTGGAGCTGCTTCTCA
*NYC1*	ACGAGCTTGAAGAGAACATACA	TCAATTGATCCAAGCTCATCCT
*HEMA1*	TCAGTGGAAAGGGTGGATGC	CTGCGTGCTCCTTTGTGAAC
*ACD1*	GTTTGTAGTGCTCGGAACTTTT	AAACGTGATCTTTGTGTACTGC
*Actin*	GATGACCCAGATCATGTTTG	GGGCGATGTAGGAAAGC

### Statistical analysis

The mean values and SD of chlorophyll contents and the expression levels of the chlorophyll biosynthesis-related gene levels were calculated on the basis of three independent biological replicates. Significant differences (*P* < 0.05) were statistically determined by Duncan’s method performed on SPSS 24.0 software, and Origin 8.0 software was used in establishing column charts (Holt, 2008; Gao, Niu & Gao, 2014).

## Results

### Plant growth analysis

**Figure 1:**
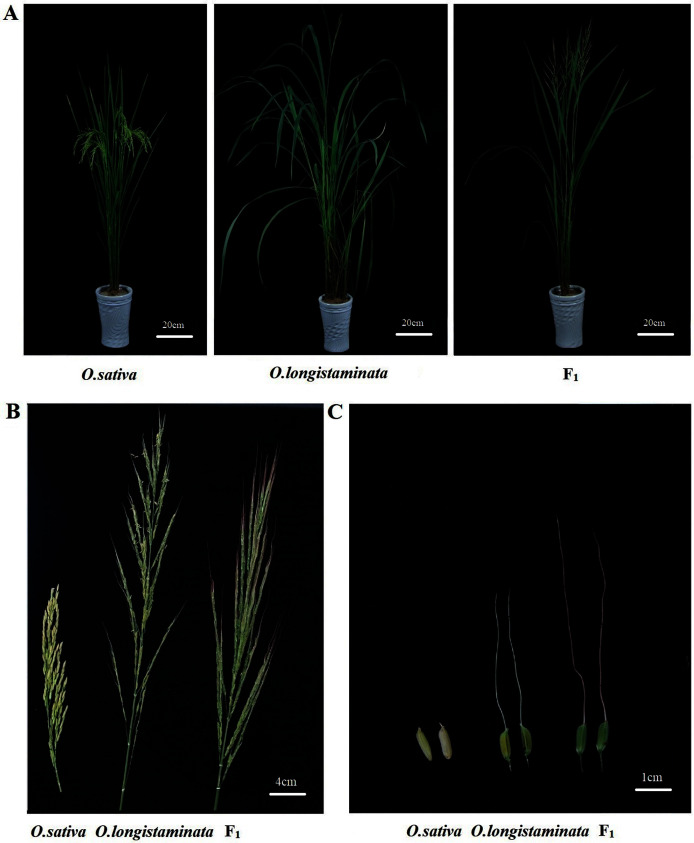
The phenotype of the three rice plants. (A) Plant; (B) panicle; (C) seed.

F_1_ hybrids were obtained from *O. sativa* (female parent) and *O. longistaminata* (male parent) *via* hybridization and embryo rescue (Yi et al., 2017). *O. sativa* is a rice cultivar ‘Huanghuazhan’ that is dwarf compared with *O. longistaminata* and their F_1_ hybrid (Fig. 1A). The panicle type of *O. sativa* was more concentrated and compact. *O. longistaminata* was up to 2 m tall, with erect but loose stalks and well-grown leaves. Compared with *O. longistaminata* and the F_1_ generation, *O. sativa* was compact and dwarf in panicle type (Fig. 1B). No awn could be found in *O. sativa* seed, but long awn could be seen in *O. longistaminata* and the F_1_ hybrid (Fig. 1C). Compared with the two parents, the F_1_ generation plants showed some different phenotypes. The height of the F_1_ generation plants was close to that of *O. longistaminata*, and the plant type was close to that of *O. sativa* with more compactness. The F_1_ generation plants also exhibited obvious long awn, similar to the male parent. However, the purple color was different from that of the male parent (Fig. 1).

### Chlorophyll contents in parents and their F_1_ generation

**Figure 2:**
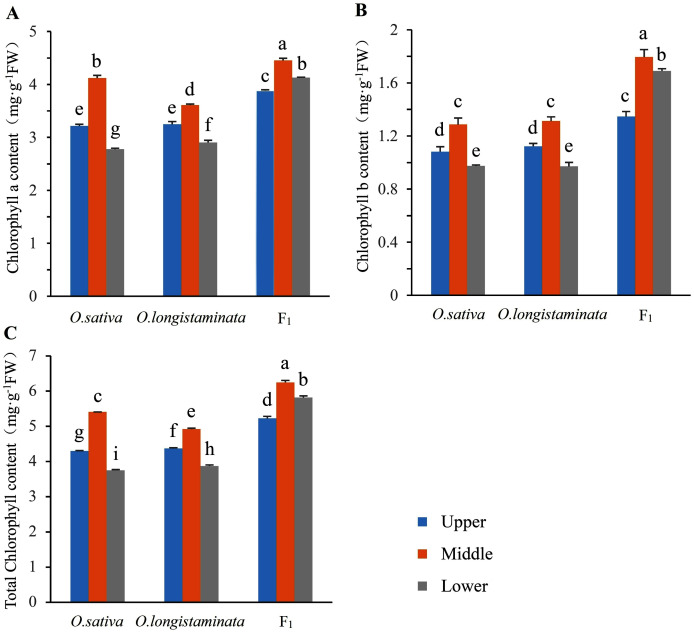
The chlorophyll contents in parents and their F_1_ generation. Error bars indicate standard deviation among three independent replicates. Data are mean ± SD of three independent replicates. The contents of chlorophyll *a, b* and total were detected by Duncan’s multiple-range test and different lowercase letters indicate significant differences at *P* < 0.05.

The chlorophyll contents in the two parents and their F_1_ generation were measured to investigate the difference in chlorophyll accumulation. The Chl *a*, Chl *b* and total Chl contents of upper-, middle-, and lower-positions of the flag leaves were detected and analyzed (Fig. 2). The Chl *a*, Chl *b*, and total Chl contents in the middle-position of the flag leaves were the highest. In *O. sativa*, the Chl *a* contents in the middle-position samples of the flag leaves were 27.95% and 48.20% higher than those in the upper- and lower-position sample at the heading stage, respectively. The Chl *b* content in the middle-position samples of the flag leaves were 19.45% and 31.63% higher than those in the upper- and lower-positions samples, respectively. In *O. longistaminata*, the Chl *a* contents in the middle position of the flag leaves were 11.08% and 24.48% higher than those in the upper- and lower-positions respectively, while the Chl *b* contents were 16.96% and 35.05%, respectively. In the F_1_ generation, the Chl *a* contents in the middle-position of the flag leaves were 14.69% and 7.75% higher than those in the upper- and lower-positions, respectively, while the Chl *b* contents were 33.33% and 6.51%, respectively. The Chl *a* and *b* and total Chl contents in the upper-, middle-, and lower-position of the flag leaves were higher in the generation than in the two parents. In particular, in the lower position of the flag leaves, the total Chl contents in F_1_ were 1.55 and 1.5 times of the contents in *O. sativa* and *O. longistaminata*, respectively.

### Photosynthetic basic parameters in parents and their F_1_ generation

Net photosynthetic rate (*Pn*), stomatal conductance (*Gs*), intercellular CO_2_ concentration (*Ci*) and transpiration rate (*Tr*) were the most important parameters reflecting the photosynthesis of plant leaves.

### Net photosynthetic rate (*Pn*)

**Figure 3:**
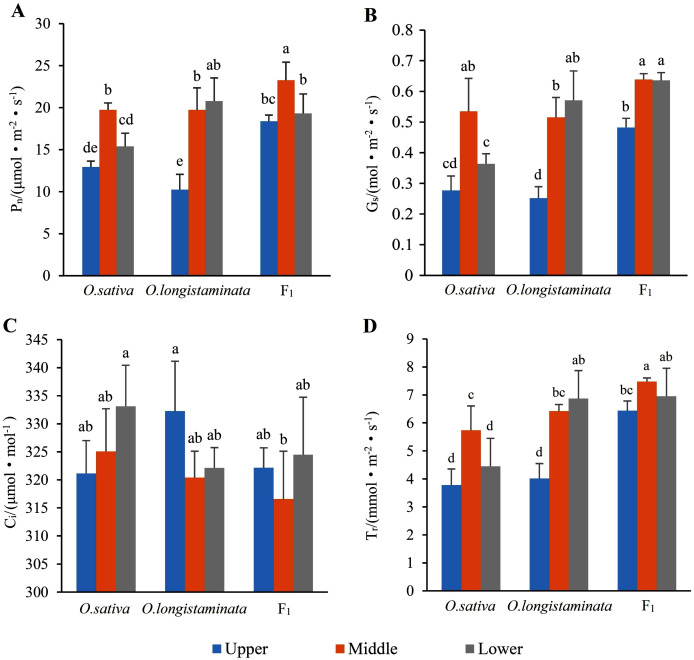
The photosynthetic parameters of flag leaf among the *O*. *sativa*, *O. longistaminata* and F_1_ generation. Error bars represent standard errors among three independent replicates. Data are the means ± SD of three replicates. The letter different indicates significant difference (*P* < 0.05)

*Pn* is the most important index reflecting plant photosynthesis. As shown in Fig. 3A, the *Pn* of F_1_ in the middle-position of the flag leaves were 1.181 and 1.180 times than that of *O. sativa* and *O. longistaminata*, respectively. In the upper- and lower-positions of the flag leaves, the *Pn* values of F_1_ were also higher than those of its parents. The *Pn* values of F_1_ and *O. sativa* were as follows: middle-position flag leaf > lower-position flag leaf > upper-position flag leaf.

### Stomatal conductance (*Gs*)

As shown in Fig. 3B, the change trends of stomatal *Gs* were similar to that of *Pn* in the flag leaves of the parents and their F_1_ generation. In the middle-position of the flag leaves, the *Gs* of F_1_ was 1.21 and 1.23 times that of *O. sativa* and *O. longistaminata*. In the upper- and lower-positions, the *Gs* of F_1_ was higher than that of its parents.

### Intercellular CO_2_ concentration (*Ci*)

As shown in Fig. 3C, in the three rice samples, *Ci* showed trends different from those of *Pn* and *Gs*. The peak *Ci* was 333.13 μmol·mol^−1^ in the lower position of the flag leaves in *O. sativa*, and the lowest *Ci* was 316.58 μmol.mol^−1^ in the middle position of the flag leaves in F_1_.

### Transpiration rate (*Tr*)

As shown in Fig. 3D, the change trends of transpiration rate (*Tr*) were similar to those of *Pn* and *Gs* in the flag leaves of the parents and their F_1_ generation. The transpiration rates of F_1_ and *O. sativa* were as follows: middle-position flag leaf > lower-position flag leaf > upper-position flag leaf. The peak value of *Tr* was 7.47 mmol·m^−2^s^−1^ in the middle-position of the flag leaves of F_1_. The lowest value of *Tr* was 3.78 mmol·m^−2^s^−1^ in the lower-position flag leaves of *O. sativa*.

### Microstructure of different positions of flag leaves

**Figure 4:**
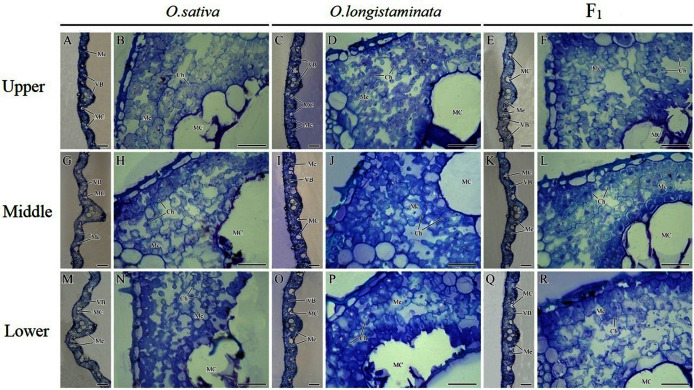
Microstructure of different positions of flag leaves. (A), (B), (G), (H), (M), (N) Transection structure of upper-, middle- and lower-positions of leaves in *O*. *sativa*; (C), (D), (I), (J), (O), (P) Transection structure of upper-, middle- and lower-positions of leaves in *O. longistaminata*; (E), (F), (K), (L), (Q), (R) Transection structure of upper-, middle- and lower-positions of leaves in F_1_. Ch, chloroplast; MC, motor cell; Me, mesophyll; VB, vascular bundle. Scale bar: (A, C, E, G, I, K, M, O, Q) = 100 μm; (B, D, F, H, J, L, N, P, R) = 20 μm.

The microstructures of the different positions of flag leaves in the three rice plants were observed, and the micrographs are shown in Fig. 4. The structure of the rice flag leaves mainly included epidermis, mesophyll cells and vascular bundles. The leaf showed several ridges of varying heights, namely leaf veins. A large vascular bundle was observed in the high ridge and a small vascular bundle was found in the low ridge. The structure of the different positions of flag leaves was basically similar, but the large vascular bundle in middle- and lower- positions was larger than that in the upper-positions (Figs. 4A, 4G, 4M). The difference in the size of large vascular bundles in leaves among the *O. sativa*, *O. longistaminata* and F_1_ were observed.

The large vascular bundle in *O. sativa* leaves was the largest (Figs. 4G, 4I, 4K, 4M, 4O and 4Q). The chloroplasts in the mesophyll cells of flag leaves were also observed. The mesophyll cell wall was folded inward, with quite large intercellular spaces. The chloroplasts were distributed around the cell wall. Differences were found in the intercellular space and chloroplast number of mesophyll cells among the rice varieties. More chloroplasts could be seen in the mesophyll cells of *O. sativa* flag leaves than in the mesophyll cells of *O. longistaminata* (Figs. 4B, 4D, 4H, 4J, 4N and 4P). The intercellular space of mesophyll cells in the upper- and lower- positions of the F_1_ flag leaves were large, while a smaller space of mesophyll cells were observed in the middle-position, along with more chloroplasts (Figs. 4F, 4L and 4R).

### Expression profiles of the genes involved in chlorophyll metabolism in different position of flag leaves

**Figure 5:**
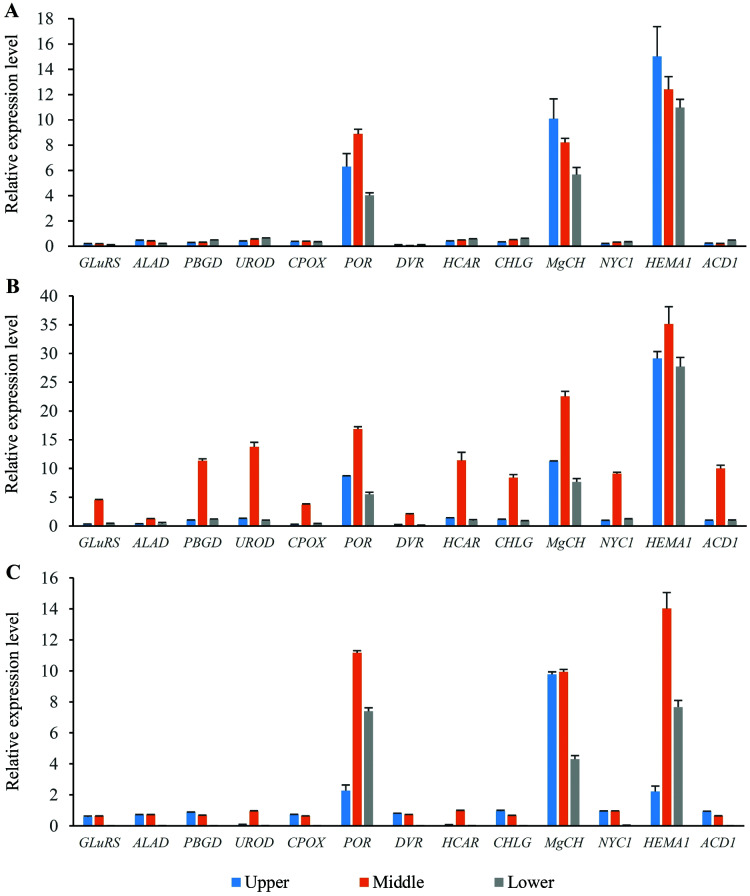
The expression levels of chlorophyll biosynthesis related genes. (A) *O*. *sativa*; (B) *O. longistaminata*; (C) F_1_. Error bars represent standard errors among three independent replicates. Data are the means ± SD of three replicates. The letter different indicates significant difference (*P* < 0.05).

Thirteen genes involved in chlorophyll metabolism were selected to elucidate the related molecular mechanism of photosynthesis in *O. sativa*, *O. longistaminata* and F_1_, and their expression profiles were determined (Fig. 5). In all the rice plants, *POR*, *MgCH*, and *HEMA1* showed higher expression levels than other genes in the flag leaves. For *HEMA1*, its expression in the middle-position of the flag leaves in *O. longistaminata* was the highest, which was 2.83 and 2.51 times that in *O. sativa* and F_1_, respectively. The *POR* and *MgCH* expression levels in the middle-position of the flag leaves in *O. longistaminata* were also the highest. For *POR*, its expression in the middle-position of the flag leaves of *O. longistaminata* was 1.90 and 1.51 times that in *O. sativa* and F_1_, respectively. The *MgCH* expression in the middle-position of the flag leaves in *O. longistaminata* was 2.75 and 2.27 times that in *O. sativa* and F_1_, respectively. In all positions of flag leaves among *O. sativa*, *O. longistaminata*, and F_1_, the expression levels of the three genes in middle-position were higher than those in the upper- or lower- positions, except *MgCH*, and *HEMA1* in *O. sativa*.

Compared with the expression levels of three genes (*POR*, *MgCH*, and *HEMA1*), those of the other 10 selected genes (*GLuRS*, *ALAD*, *PBGD*, *UROD*, *CPOX*, *DVR*, *HCAR*, *CHLG*, *NYC1* and *ACD1*) were relatively low. In the female parent (*O. sativa*) and F_1_ generation, the expression of these 10 genes was lower than that in the male parent (*O. longistaminata*). For *DVR*, its expression in the lower-position of the flag leaves in F_1_ was the lowest, which was 93.16% and 95.06% lower than that in *O. sativa* and *O. longistaminata*, respectively.

## Discussion

At present, *O. longistaminata* has been widely used in rice breeding. Numerous results about the disease resistance and agronomic characters of *O. longistaminata* were reported. *XA21*, a gene resistant to bacterial blight, has been identified and cloned by map-based cloning (Song et al., 1995; Hu et al., 2003). Xu and his group mapped a rice blast resistance gene (*Pi57t*) on chromosome 12 with SSR markers (Xu et al., 2015). *O. longistaminata* also has many unique agronomic characteristics, such as long anther, large stigma, and purple glume, and so on, which could be used to improve the quality and yield of cultivated rice. In 2010, QTL analysis was carried out in F_2_ generation constructed by the introduction line and the maintainer line of *O. longistaminata*, and *qPES-9* were detected to control the total and bilateral stigma exposure rate. In a backcross population constructed from *O. longistaminata* and RD23, the gene controlling the color of purple styma was located on rice’s chromosome 6, which may be the allele of *PS4(t)* regulating the color of styma in cultivated rice (Zhao et al., 2012). *S40* locus was identified using gene expression and genomic sequence analysis, and it was related neutral allele in rice breeding (Chen et al., 2017). In the present study, the F_1_ generation demonstrated higher height, more compact plant type and longer awn than its parents, which showed differences among those characteristics. The purple color of the long awn was an obvious characteristic. The obvious difference between the F_1_ generation and its parents provided some reference for future breeding using *O. longistaminata*.

In higher plants, photosynthesis is the main method to biosynthesize organic matter. Chlorophyll could capture light energy and transform energy, and it is necessary for the formation and stability of light-capturing chlorophyll *a/b* protein complex (Reinsberg et al., 2001; Jiang et al., 2014). The chlorophyll content of higher plants decreased under adverse environment, which may be due to slower chlorophyll biosynthesis, faster degradation processes, or both (Mohanty, Grimm & Tripathy, 2006; Huang et al., 2017). Lower chlorophyll content will reduce the photosynthetic rate, leading to slow plant production or even death, and seriously affect the yield of crops. Chlorophyll synthesis and degradation are very important biochemical processes in plants (Eckhardt, Grimm & Hörtensteiner, 2004). Numerous of biotic and abiotic stresses affect biomass and yield throughout the plant life cycle (Yamaguchi-Shinozaki & Shinozaki, 2006; Li et al., 2019; Que et al., 2019; Salvi et al., 2021).

Photosynthesis is the basis of rice plant growth, development and morphogenesis. It can provide materials and energy for rice plant growth. Rice yield is mainly determined by photosynthetic area, photosynthetic time, photosynthetic rate, source, storage-capacity and flow differently. The F_1_ generation showed higher contents of Chl *a*, Chl *b* and total Chl in the upper-, middle- and lower-positions of its flag leaves than its parents. Heterosis describes a biological phenomenon in which a hybrid generation is superior to its parent in terms of size, growth rate, fecundity, tolerance, and viability. It is a common phenomenon in higher plants, and it has been widely used in rice breeding. It has become an essential tool to improve the quality and yield of rice. Under the condition of high light intensity, the chlorophyll content was positively correlated with the *Pn* of the flag leaf in rice. The F_1_ generation plants in rice are better than their parents in terms of yield resistance to disease, insect resistance and stress resistance (Hochholdinger & Hoecker, 2007; Baranwal et al., 2012; Goff & Zhang, 2013; Fan et al., 2019).

In this study, the microstructure results showed that the flag leaves of F_1_ generation have large mesophyll cell gaps in the upper- and lower-positions and small mesophyll cell gaps in the middle-position, with more chloroplasts. The high chlorophyll content of F_1_ may be related to heterosis, and may provide some reference for future application. Net photosynthetic rate (*Pn*), stomatal conductance (*Gs*), intercellular CO_2_ concentration (*Ci*) and transpiration rate (*Tr*) are the four important indices reflecting plant photosynthesis. In this study, the net photosynthetic rates (*Pn*), stomatal conductance (*Gs*) and transpiration rate (*Tr*) of the F_1_ generation were higher than those of the two parents. The intercellular CO_2_ concentration (*Ci*) was lower than that of the two parents, especially in the middle-position of the flag leaves. The results possibly suggested the heterosis of F_1_ generation compared with its two parents.

Chlorophyll biosynthesis and accumulation are a complex and dynamic biological process that involves the coordination of multiple genes. In higher plants, chlorophyll biosynthesis occurs in the chloroplasts. The mechanism of chlorophyll biosynthesis and degradation is well understood (Eckhardt, Grimm & Hörtensteiner, 2004; Tanaka & Tanaka, 2006, 2007). In previous studies, the biosynthesis pathway of Chlorophyll has been reported in many plants, such as rice (Chatterjee & Kundu, 2015), Arabidopsis (Bailey et al., 2001; Meskauskiene et al., 2001), *Oenanthe* water drop (Jiang et al., 2014), and celery (Huang et al., 2017). The universal tetrapyrrole precursor of Chlorophyll was synthesized by Glutamyl-tRNA reductase (GluTR) and GSA aminotransferase (GSA-AT). ALA dehydratase (ALAD) condensed two ALA molecules into pyrrole. With PBG deaminase (PBGD), porphobilinogen (PBG) and four molecules of PBG were polymerized into 1-hydroxymethylbillane (HMB), a linear tetrapyrrole (Stenbaek et al., 1995). Then, uroporphyrinogen III synthase (UROS), uroporphyrinogen III decarboxylase (UROD), Oxygen-dependent coproporhyrinogen III oxidase; (COPX), protoporphyrinogen IX oxidase (PPOX), Mg-chelatase I-subunit (CHLI), Mg-protoporphyrin IX methyltransferase; (CHLM), Mg-protoporphyrin monomethyl ester cyclase (CHL27), protochlorophyllide oxidoreductase (POR) involved into the subsequent chlorophyll biosynthesis (Eckhardt, Grimm & Hörtensteiner, 2004; Tanaka & Tanaka, 2007; Stenbaek et al., 1995). Here, *POR*, *MgCH*, and *HEMA1* genes showed increased expression levels in the female parent *O. sativa*, male parent *O. longistaminata* and their F_1_ generation. These levels were the highest in *O. longistaminata*. The *HEMA1* encoding enzyme played key roles in the initiation of chlorophyll biosynthesis. Mg-protoporphyrin was catalyzed by magnesium chelatase (MgCH), which is also one of the key enzymes in chlorophyll biosynthesis. The *POR* encoding the protochlorophyllide oxidoreductase play an important role in the later stage of chlorophyll biosynthesis. (Eckhardt, Grimm & Hörtensteiner, 2004; Tanaka & Tanaka, 2007; Stenbaek et al., 1995) The three genes maybe play important roles in the chlorophyll biosynthesis of *O. longistaminata* and its descendant. This finding needs to be verified by further molecular intervention.

## Conclusion

In this study, the chlorophyll contents and photosynthetic parameters in the different positions of the flag leaves among *O. longistaminata*, *O. sativa* and their F_1_ generation were determined and analyzed. The expression profiles of chlorophyll biosynthesis-related genes were also detected. F_1_ showed an obvious long awn with purple color. The chlorophyll content of F_1_ was higher than that of the two parents. *POR*, *MgCH* and *HEMA1* may play important roles in the chlorophyll biosynthesis of F_1_ generation. This study provided a potential reference for studying the photosynthesis and heterosis utilization of African wild rice and cultivated rice.

## Supplemental Information

10.7717/peerj.12099/supp-1 Supplemental Information 1 The chlorophyll contents in parents and their F_1_ generation.

10.7717/peerj.12099/supp-2 Supplemental Information 2 The photosynthetic parameters of flag leaf among the HHZ, *O. longistaminata* and F_1_ generation.

10.7717/peerj.12099/supp-3 Supplemental Information 3 The expression levels of chlorophyll biosynthesis related genes.

## Additional Information and Declarations

### Data Availability

The following information was supplied regarding data availability: The raw data are available in the Supplemental Files.
